# Evaluation of the models generated from clinical features and deep learning-based segmentations: Can thoracic CT on admission help us to predict hospitalized COVID-19 patients who will require intensive care?

**DOI:** 10.1186/s12880-022-00833-2

**Published:** 2022-06-07

**Authors:** Mutlu Gülbay, Aliye Baştuğ, Erdem Özkan, Büşra Yüce Öztürk, Bökebatur Ahmet Raşit Mendi, Hürrem Bodur

**Affiliations:** 1 Department of Radiology, Ankara City Hospital, Üniversiteler Mahallesi 1604. Cadde No: 9, 06800 Çankaya, Ankara, Turkey; 2 Department of Infectious Diseases and Clinical Microbiology, University of Health Sciences Turkey, Gülhane Faculty of Medicine, Ankara City Hospital, Ankara, Turkey; 3 Department of Clinical Microbiology and Infectious Diseases, Ankara City Hospital, Ankara, Turkey

**Keywords:** COVID-19, Deep learning, Artificial intelligence, Computed tomography, Radiomics, Machine learning, Logistic regression models

## Abstract

**Background:**

The aim of the study was to predict the probability of intensive care unit (ICU) care for inpatient COVID-19 cases using clinical and artificial intelligence segmentation-based volumetric and CT-radiomics parameters on admission.

**Methods:**

Twenty-eight clinical/laboratory features, 21 volumetric parameters, and 74 radiomics parameters obtained by deep learning (DL)-based segmentations from CT examinations of 191 severe COVID-19 inpatients admitted between March 2020 and March 2021 were collected. Patients were divided into Group 1 (117 patients discharged from the inpatient service) and Group 2 (74 patients transferred to the ICU), and the differences between the groups were evaluated with the T-test and Mann–Whitney test. The sensitivities and specificities of significantly different parameters were evaluated by ROC analysis. Subsequently, 152 (79.5%) patients were assigned to the training/cross-validation set, and 39 (20.5%) patients were assigned to the test set. Clinical, radiological, and combined logit-fit models were generated by using the Bayesian information criterion from the training set and optimized via tenfold cross-validation. To simultaneously use all of the clinical, volumetric, and radiomics parameters, a random forest model was produced, and this model was trained by using a balanced training set created by adding synthetic data to the existing training/cross-validation set. The results of the models in predicting ICU patients were evaluated with the test set.

**Results:**

No parameter individually created a reliable classifier. When the test set was evaluated with the final models, the AUC values were 0.736, 0.708, and 0.794, the specificity values were 79.17%, 79.17%, and 87.50%, the sensitivity values were 66.67%, 60%, and 73.33%, and the F1 values were 0.67, 0.62, and 0.76 for the clinical, radiological, and combined logit-fit models, respectively. The random forest model that was trained with the balanced training/cross-validation set was the most successful model, achieving an AUC of 0.837, specificity of 87.50%, sensitivity of 80%, and F1 value of 0.80 in the test set.

**Conclusion:**

By using a machine learning algorithm that was composed of clinical and DL-segmentation-based radiological parameters and that was trained with a balanced data set, COVID-19 patients who may require intensive care could be successfully predicted.

## Background

Severe COVID-19 patients who are admitted to the inpatient ward due to the need for supplemental oxygen or due to evidence of systemic inflammation must be monitored for the development of critical illness, a rapid increase in oxygen needs and/or an increasing systemic deterioration [1]. For patients who progress to a critical illness level, transfers to the intensive care unit (ICU) are required; additionally, depending on the severity of the condition, the patient may also need oxygen delivery through a high-flow device, noninvasive ventilation, invasive mechanical ventilation, or extracorporeal membrane oxygenation [1]. Planning the ICU bed capacity is of primary importance during pandemic surges [2] since limitations in the ICU bed capacity have been reported to have an effect on mortality [3]. Thus, it is important to predict the need for ICUs, especially for patients with a severe clinical condition that requires inpatient treatment [4, 5]. Additionally, starting remdesivir in the ward was recommended if disease progression was predicted [1].

Although models for identifying ICU candidate patients have been reported, most of these models are based only on clinical data [6–11]. In a study where the candidate parameters included the presence or absence of chest X-ray findings, it was noted that this parameter was not included in the final model [12]. Promising results were achieved by combining the clinical data with the semiquantitative visual severity scores (VSS) depending on the volume, type, and extent of the infiltration that were measured on chest X-ray and CT [13–15].

Radiomics analysis extracts different quantitative data from medical images with various algorithms, and these data are used in further analyses for decision support [16]. Studies using radiomics models and machine learning methods have shown that these methods can diagnose COVID-19 [17, 18] and can determine its prognosis [19]. Although combined models of the clinical and radiomics parameters in RT-PCR-positive cohorts were reported [20], studies evaluating the efficacy of models that include clinical, quantitative volumetric and radiomics parameters for predicting disease progression in hospitalized COVID-19 patients are lacking.

Using deep learning (DL) for a COVID-19 diagnosis was previously studied by using chest X-ray and CT parameters in pretrained or customized models, and the results were successful [21]. DL networks are also used for automated segmentation, and a high accuracy was shown in the U-Net architecture for the CT images in COVID-19 patients [22].

The aim of this study was to generate and compare models that predict the need for ICUs in hospitalized COVID-19 patients using clinical features and volumetric and radiomics data that were calculated by automated segmentations.

## Materials and methods

This retrospective, cross-sectional, single-center study was approved by our institution’s review board (EK-E1-21-2090), and written informed consent was waived. All of the procedures that were performed in this study were in accordance with the 1964 Helsinki Declaration and its later amendments.

## Study population

**Fig. 1:**
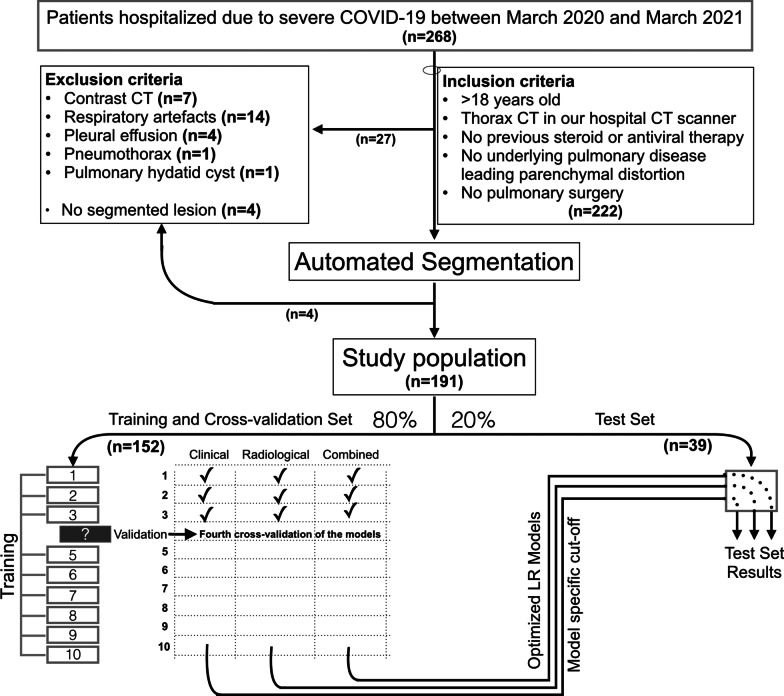
Flowchart of the study. Clinical, Radiological and Combined models are the final models in cross-validation. LR is Logistic regression

A total of 268 RT-PCR-positive severe COVID-19 patients hospitalized consecutively in our inpatient ward between March 2020 and March 2021 were evaluated (Fig. 1). All these patients had one or more of the criteria for *severe illness* [1]: an SpO_2_ < 94% when breathing room air, a < 300 mmHg arterial partial oxygen pressure to fraction of inspired oxygen (PaO_2_/FiO_2_) ratio, a respiratory rate > 30 per minute or infection involving more than 50% of the lung parenchyma. Patients were transferred to the ICU when one or more of the signs of *critical illness* [1], including acute respiratory distress syndrome, septic shock, and multiorgan failure, had developed.

The inclusion criteria in this patient group were patients older than 18 years of age, patients who had a thoracic CT scan in our hospital, patients who did not receive any steroid or antiviral treatment before the CT study, patients with no interstitial pulmonary disease, and patients who had not undergone pulmonary surgery, and 222 patients met these criteria. In this group, patients with enhanced CT examinations (n = 7, all were suspicious for embolism), respiratory artifacts (n = 14), massive pleural effusion (over two-thirds of the hemithorax) (n = 4), pneumothorax (n = 1), and cystic lung disease (n = 1) were excluded from the study.

### CT protocol

CT studies were performed with a 128-detector system (GE Revolution, General Electric, Milwaukee, WI) from the first rib to the adrenal glands, nonenhanced by using the following parameters: 100 kV, 110 mAs, body filter, a 1.25 mm slice thickness, a 512 × 512 reconstruction matrix, a spiral pitch factor of 1.375:1, BonePlus convolution kernel, adaptive statistical iterative reconstruction of 70%.

### Deep learning segmentation and radiomics feature calculation

The entire lung parenchyma and pneumonic lesions were segmented by using Quibim’s U-Net model, which is a convolutional neural network architecture that uses the ResNet-34 backbone, which was developed for the ‘A European initiative for automated diagnosis and quantitative analysis of COVID-19 on imaging’ project.

Slices of the studies were preprocessed as the segmentation model input by applying a constant lung window level (WW = 1600, WL = − 600), normalization in range [0, 1], as well as by using the Balance Contrast Enhancement Technique (BCET preprocessing). Thus, the basic shapes of the image histograms were maintained.

Several metrics were accounted for in order to evaluate the segmentation model. Dice Similarity Coefficient (DSC) and Intersection over Union (IoU) values were calculated on all of the scans with at least 1000 voxels in the ground truth segmentation. Due to the fact that DSC and IoU are zero in cases without ground truth mask, the final test set for these metrics was determined by using a histogram-based threshold of more than 1000 positive voxels. Average false positive and false negative volumes were calculated for all of the scans. In addition, Pearson's correlation coefficients of positive prediction and ground truth were determined.

Two authors (MG, 16 years of experience and EO, last year of residency training) checked whether all ground glass opacities (GGO), consolidation or crazy paving areas in the CT studies were segmented by the DL algorithm. It was noted that DL did not segment GGO that were smaller than 1 mL, and patients who only had such lesions were excluded from the study (n = 4). Finally, the final study population consisted of 191 patients.

The radiomics features were calculated using Quibim Texture Analysis software (Quibim SL, Valencia, Spain) from the obtained segmentations by the following parameters: (1) Resampled voxel size 1 × 1 × 1 mm^3^ by using bicubic interpolation, (2) Fixed bin-width of 25 for gray value discretization, (3) Density normalization according to Eq. (1):

1 $$f\left( x \right) = \frac{{\left( {x - \mu_{x} } \right)}}{{\sigma_{x} }} \times S$$

where f(x) is the normalized voxel density, x is the original density, μ_x_ is the mean density, σ_x_ is the standard deviation and S is the scaling factor (set to 500). (4) A voxel array shift of 1024 was added to prevent the negative values from being squared. (5) Second-order matrices were calculated using a distance of 1 voxel and 13 isotropic displacement vectors at angles of 0°, 45°, 90° and 135°.

### Statistical analysis

Patients were categorized into Group 1 (patients who recovered with treatment in the inpatient ward) and Group 2 (patients transferred to the ICU for progressive disease from the inpatient ward).

The data obtained from the patients were divided into (1) clinical data consisting of the demographic data of the patients, comorbid disease history, therapeutics given to the patient in the inpatient service, oxygen saturation, complete blood count, biochemical parameters, and acute phase reactants obtained at admission and (2) radiological data consisting of the volumetric data of the whole lung, the inflamed lung parenchyma as segmented by DL and the first- and second-order radiomics parameters calculated from the segmented lesions.

Comparison of the nominal data between the two groups was performed with the chi-squared test or the Fisher's exact test. For continuous data, the values of a normally distributed parameter were given as the mean ± SD, and the values of nonnormally distributed parameters were provided as the median (IQR). Comparisons of the groups were conducted with the T-test or Mann–Whitney test, accordingly.

If a parameter differed significantly between the two groups, the area under the curve (AUC) was calculated with the receiver operator characteristic (ROC) test, and the cutoff value, optimal sensitivity, and specificity were determined by using the Youden index. Logistic regression was used for the univariate nominal parameters to calculate the sensitivity and specificity.

After the patient population was randomly divided into a training and cross-validation set (n = 152, 79.6%) and a test set (n = 39, 20.4%), logit fit models were created by using the Bayesian information criterion (BIC) from the training set. The clinical model was selected from the clinical data, and the radiological model was selected from the radiological data. A combined model from both clinical and radiological data was also constructed. The adequacy of the model’s parameters in predicting the categorical outcomes was evaluated with the Hosmer–Lemeshow goodness-of-fit test. Multicollinearity was evaluated by calculating the variance inflation factor (VIF).

Models were optimized by calculating cost function (log loss), and the gradient descent optimization algorithm and initial theta vectors were replaced with the optimized ones. By using a tenfold cross-validation, the mean sensitivity, specificity, and accuracy values of the model were calculated by averaging all of the cross-validation results, and the model-specific cutoff values for each model were calculated via the Youden index of ROC analyses [23]. The test set results were obtained by using optimized models and model-specific cutoff values. The C-index and 95% CI values of the models were further separately calculated for the training and cross-validation sets via 1000 bootstrapping studies.

To solve the class imbalance problem, the Synthetic Minority Oversampling Technique (SMOTE) algorithm was used by using the “smotefamily” package in the R statistical computing environment (R Foundation for Statistical Computing, Vienna, Austria) [24]. During the generation of the synthetic data, k = 3 was selected for the K-nearest neighbor algorithm.

Another model including all of the clinical and radiological parameters in the study was created via the random forest classification algorithm, and this model was trained with the balanced training set containing the synthetic data. The effectiveness of the final random forest model was evaluated with the same test set that was used for the logit-fit models.

Statistical analyses were performed using IBM SPSS v23 (IBM Corp, Armonk, NY), MedCalc v20.011 (MedCalc Software bvba, Ostend, Belgium), R v4.0.2 (R foundation, Vienna, Austria) and XLStat statistical and data analysis v2021.3.1 (Addinsoft, NY, USA). The power analysis was conducted using G*Power 3.1.

## Results

### Group features, demographics, symptoms and findings

There were 117 patients in Group 1 (61.6%) and 74 in Group 2 (38.4%). The mean age of the patients was 65.45 ± 14.02 (26–96 years), 57.6% of the patients were male, and 42.4% were female. ICU patients were followed in the ward for an average of 3.2 days (1–12 days) prior to transfer to the ICU. Whereas Group 1 patients were discharged with a mean duration of 8.8 ± 4.7 days (2–29 days), Group 2 patients had a mean duration of 19.2 ± 13.8 days (7–41 days, including ICU stay) of hospitalization that resulted in either death (n = 3) or discharge (n = 71).

The mean age of the patients and the number of males were higher in Group 2, and the differences were significant (Table 1). Regarding the symptoms and findings, only fever was significantly different between the two groups. Among the comorbidities, patients diagnosed with chronic renal failure or coronary heart disease required significantly more ICU admissions (Table 1). Patients who needed corticosteroids in the ward were more frequently transferred to the ICU (Table 1).

**Table 1:** Demographics, symptoms, findings, comparison between the groups and discrimination assessment

Parameter	Group 1	Group 2	*p*	Cut-off or Risk factor	Sensitivity %	Specificity %	AUC
Age (years)	62 (21)	70 (23.3)	< 0.001^a^	> 71	55.9	73.1	0.679^d^
Sex	Male: 52.0%	Male: 48.0%	0.005^b^	Male	67.3	50.3	0.774^e^
	Female: 69.3%	Female: 30.7%					
Fever (body temperature > 38 °C)	58.0%	72.0%	0.014^b^	Present	72.0	42.0	0.770^e^
Dyspnea	31.9%	43.8%	0.082^b^				
Cough	49.5%	50.5%	0.491^b^				
Diabetes mellitus	28.7%	34.6%	0.344^b^				
Hypertension	42.5%	57.5%	0.210^b^				
Acute renal failure	1.9%	3.7%	0.446^c^				
Chronic renal failure	0.4%	3.8%	0.001^c^	Present	9.4	99.3	0.863^e^
Coronary heart disease	21.0%	33.6%	0.022^b^	Present	33.6	79.0	0.764^e^
COPD	15.3%	15.0%	0.941^b^				
Malignity	3.2%	6.5%	0.199^b^				
CS treatment at the ward	R: 66.7%	R: 83.8%	0.009^b^	Need for CS treatment	83.78	33.33	0.595^e^
	N/R: 33.3%	N/R: 16.2%					
High dose CS pulse treatment at the ward	R: 30.9%	R: 63.8%	< 0.001^b^	Need for pulse CS treatment	63.77	69.07	0.680^e^
	N/R: 69.1%	N/R:36.2%					

Group results were given as Median (IQR)

Group 1: Patients discharged from inpatient floor; Group 2: Patients transferred to ICU

*COPD* chronic obstructive pulmonary disease, *CS* corticosteroid, *R* received, *N/R* not received

^a^Mann–Whitney test result

^b^Chi-squared test result

^c^Fisher’s exact test result

^d^ROC analysis results

^e^One-parameter logistic regression result

### Laboratory findings

Most of the laboratory findings differed significantly between the two groups (Table 2). However, at the time of admission, it was observed that patients who needed ICU care did not have a lower oxygen saturation value. Among the blood tests, procalcitonin was the most effective univariate classifier (Table 2).

**Table 2:** Laboratory findings, comparison between the groups and results of ROC analysis

Parameter	Group 1	Group 2	*p*^a^	Cut-off	Sensitivity %	Specificity %	AUC^b^
SPO_2_ (%)	92 (2)	92 (4)	0.987				
wbc count (/μL)	6100 (2730)	5860 (3320)	0.948				
Lymhoocyte count (/μL)	1100 (670)	810 (530)	0.0002	≤ 1000	72.0	57.3	0.655
Neutrophil/Lymphocyte ratio	5.14 (4.07)	8.59 (8.30)	0.0001	> 3.65	75.7	49.6	0.659
Platelet count (× 10^3^/μL)	225 (93.5)	192 (96)	0.010	≤ 214	61.7	56.0	0.593
eGFR (mL/min/1.73 m^2^)	87 (33)	69 (50)	0.0003	≤ 74	56.1	66.2	0.632
Alanine aminotransferase (ALT; Units/L)	28 (26)	29 (28)	0.594				
Aspartate aminotransferase (AST; Units/L)	34 (21)	44 (39)	0.0004	> 37	68.2	61.1	0.668
Lactate dehydrogenase (LDH; Units/L)	294 (120)	390 (182)	0.00001	> 360	66.4	75.8	0.755
Creatine kinase (CK; Units/L)	99.5 (110)	179 (247)	0.0001	> 172	53.3	76.3	0.676
C-reactive protein (CRP; mg/L)	71 (85.5)	100 (104)	0.0005	> 134	39.3	86.0	0.627
Procalcitonin (PCT; μg/L)	0.04 (0.06)	0.17 (0.27)	0.00001	> 0.11	62.6	81.5	0.785
Ferritin (ng/mL)	254 (407)	445 (706)	0.0001	> 408	58.3	66.7	0.648
D-Dimer (ng/mL)	0.70 (0.60)	1.00 (1.28)	0.001	> 0.92	51.9	69.3	0.626

Group results were given as Median (IQR)

Group 1: Patients discharged from inpatient floor; Group 2: Patients transferred to ICU

*SPO*_*2*_ Pulse oximetry saturation, *eGFR* estimated-glomerular filtration rate

^a^Mann–Whitney test result

^b^ROC analysis result

### DL segmentation findings and radiomics

The time between the onset of symptoms and the CT examination was 6 [7] days in Group 1 and 5 [5] days in Group 2 (*p* = 0.775, Mann–Whitney test). The positive RT-PCR test result and the CT study were conducted on the same day.

The DL algorithm segmented both the whole lung tissue and the pneumonic areas of COVID-19 infection in the patients. In Group 2 patients, both the percentage and volume of pneumonic tissue secondary to COVID-19 were significantly higher (Table 3) than those in Group 1 patients. Additionally, in Group 2, the mean total lung volume was decreased by 11.2% compared to that in Group 1.

**Table 3:** Volumetric data, comparison between the groups and results of ROC analysis

Parameter	Group 1	Group 2	*p*	Cut-off	Sensitivity %	Specificity %	AUC^c^
*Right lung*							
Upper Third (%)	5 (16)	11.5 (20)	0.011^a^	> 9	59.5	65.8	0.608
Middle Third (%)	14 (17)	21.5 (27)	0.015^a^	> 24	48.7	77.8	0.604
Lower Third (%)	12 (18)	18.5 (30)	0.012^a^	> 35	26.4	93.2	0.608
Infected Total (%)	12 (12)	21 (21)	0.002^a^	> 19	54.1	76.1	0.635
Upper Third (mL)	25 (71)	51 (105)	0.032^a^	> 39	60.1	60.1	0.592
Middle Third (mL)	136 (152)	174.5 (220)	0.120^a^				
Lower Third (mL)	41 (69)	63.5 (94)	0.049^a^	> 77	46.0	73.0	0.584
Infected Total (mL)	222 (235)	307.5 (416)	0.010^a^	> 354	47.3	76.9	0.611
Whole Right Lung Volume(mL)	1849 ± 574	1628 ± 515	0.008^b^	≤ 1516	46.0	69.2	0.592
*Left lung*							
Upper Third (%)	1 (7)	4 (16)	0.020^a^	> 7	40.5	77.8	0.597
Middle Third (%)	10 (20)	19 (30)	0.005^a^	> 12	66.2	58.1	0.622
Lower Third (%)	11 (22)	21 (31)	0.024^a^	> 26	43.2	76.1	0.597
Infected Total (%)	10 (16)	15.5 (19)	0.001^a^	> 11	63.5	59.8	0.640
Upper Third (mL)	9 (30)	13.5 (79)	0.012^a^	> 71	27.0	92.3	0.575
Middle Third (mL)	73 (136)	125 (174)	0.080^a^				
Lower Third (mL)	38 (70)	67 (88)	0.060^a^				
Infected Total (mL)	139 (209)	232 (281)	0.092^a^				
Whole Left Lung Volume(mL)	1583 ± 525	1419 ± 491	0.033^b^	≤ 1819	83.8	31.6	0.581
*Both lungs*							
Total infected lung (%)	12.26 (12.55)	18.60 (19.24)	< 0.001^a^	> 16.61	59.5%	70.1%	0.674
Total infected lung (mL)	377 (441)	583.5 (612)	0.001^a^	> 435	68.9%	58.1%	0.638
Total Lung Volume	3433 ± 1078	3049 ± 956	0.011^b^	≤ 3804	83.8%	33.3%	0.589

Group results were given as Median (IQR) or Mean ± SD according to the parameter’s distribution

Group 1: Patients discharged from inpatient floor; Group 2: Patients transferred to ICU

^a^Mann–Whitney test result

^b^T-test result

^c^ROC analysis result

Eighteen first-order and 58 second-order radiomics parameters were calculated from the segmentations (Table 4). The skewness was higher in Group 1, and the mean density was higher in Group 2 (Table 4).

**Table 4:** Radiomic parameters, comparison between the groups and results of ROC analysis

Parameter	Group 1	Group 2	*p*	Cut-off	Sensitivity %	Specificity %	AUC^c^
*First order*							
10th percentile*	− 126.15 (109.66)	− 86.95 (113.60)	0.010^a^	> − 87.04	50.0	73.5	0.611
90th percentile*	508.17 ± 147.39	591.50 ± 120.42	< 0.001^b^	> 488.26	85.1	45.3	0.672
Energy (× 10^11^)	5.71 (6.94)	11.4 (9.94)	< 0.001^a^	> 6.90	66.2	65.8	0.675
Entropy	5.15 (0.32)	5.29 (0.20)	< 0.001^a^	> 5.25	66.2	65.0	0.672
Interquartile Range*	355.01 ± 93.75	398.27 ± 84.74	0.001^a^	> 319.42	87.8	40.2	0.644
Kurtosis	2.64 (1.17)	2.26 (0.63)	< 0.001^a^	≤ 2.56	74.3	53.0	0.652
Maximum*	998.36 ± 122.10	1009.82 ± 121.38	0.527^b^				
Mean Absolute Deviation*	199.58 ± 36.68	214.74 ± 32.06	0.003^b^	> 201.11	75.7	50.4	0.629
Mean*	162.99 ± 119.13	243.10 ± 122.06	< 0.001^b^	> 159.23	77.0	54.7	0.691
Median*	129.73 ± 109.82	231.95 ± 146.89	0.001^b^	> 185.67	60.8	71.8	0.702
Minimum*	− 479.49 ± 83.67	− 487.67 ± 89.95	0.506^b^				
Range*	1477.85 ± 101.11	1497.49 ± 134.92	0.278^b^				
RMAD *	146.78 ± 35.51	162.46 ± 31.45	0.002^b^	> 134.00	86.5	39.3	0.636
Root Mean Squared*	1211.68 ± 121.50	1293.10 ± 121.19	0.001^b^	> 1213.80	74.3	57.3	0.693
Skewness	0.578 (0.730)	0.187 (0.648)	< 0.001^a^	≤ 0.788	94.6	39.3	0.721
Total Energy (× 10^11^)	7.09 (6.95)	10.00 (9.94)	< 0.001^a^	> 6.9	66.2	65.8	0.675
Uniformity	0.0323 (0.0100)	0.0281 (0.0058)	< 0.001^a^	≤ 0.0292	62.2	67,5	0.678
Variance	59,791 ± 17,619	66,375 ± 15,803	0.010^b^	> 59,734	73.0	53.0	0.618
*Second order—gray level cooccurrence matrix (GLCM)*							
Autocorrelation	721.06 (420.12)	1009.76 (446.41)	< 0.001^a^	> 706.42	87.8	47.9	0.698
Cluster tendency	310.78 (144.06)	354.96 (89.13)	0.012^a^	> 311.05	75.7	51.3	0.608
Clustershade	2585.01 (3335.90)	807.02 (4056,87)	< 0.001^a^	≤ 1429.10	60.8	70.1	0.676
Maximum probability	0.00464 (0.00345)	0.00353 (0.00332)	0.005^a^	≤ 0.00330	47.3	73.5	0.620
Sumentropy	6.0181 (0.3201)	6.1410 (0.1770)	< 0.001^a^	> 6.0286	78.4	53.9	0.666
Joint energy (× 10^–3^)	1.628 (1.022)	1.320 (0.701)	0.002^a^	≤ 1.600	71.6	53.0	0.631
Joint entropy	9.814 (0.514)	10.016 (0.481)	0.005^a^	> 9.9209	62.2	62.4	0.620
Correlation	0.6750 ± 0.0810	0.6906 ± 0.0844	0.206^b^				
Contrast	58.517 (24.270)	61.059 (24.522)	0.203^a^				
IDN	0.9190 ± 0.0168	0.9169 ± 0.0116	0.238^b^				
IMC	0.7626 (0.1008)	0.7949 (0.1031)	0.027^a^	> 0.8311	35.1	85.5	0.596
*Second order—gray level run length matrix (GLRLM)*							
Gray Level NUN (× 10^–2^)	3.202 (0.992)	2.776 (0.577)	< 0.001^a^	≤ 2.907	63.5	66.7	0.680
Gray Level Variance	94.812 (39.683)	107.492 (25.276)	0.007^a^	> 96.082	73.0	53.0	0.617
High GL Run Emphasis	751.038 (415.427)	1021.780 (440.303)	< 0.001^a^	< 724.968	89.2	47.9	0.699
Long-run emphasis	1.192 (0.052)	1.188 (0.058)	0.465^a^				
Long-run High GLE	882.784 (474.590)	1241.118 (520.097)	< 0.001^a^	> 1052.402	68.9	67.5	0.704
Long-run Low GLE(× 10^–3^)	2.889 (1.573)	2.103 (1.366)	< 0.001^a^	≤ 2.504	67.6	64.1	0.667
Low GL Run Emphasis (× 10^–3^)	2.402 (1.146)	1.802 (1.043)	< 0.001^a^	≤ 2.107	68.9	63.2	0.673
Run Entropy	5.501 (0.253)	5.618 (0.171)	< 0.001^a^	> 5.510	78.4	53.0	0.686
Run Length Nonuniformity	0.892 (0.240)	0.895 (0.269)	0.478^a^				
Run Percentage	0.942 (0.138)	0.943 (0.154)	0.473^a^				
Run Variance	0.660 (0.183)	0.646 (0.222)	0.455^a^				
Short Run Emphasis	0.957 (0.103)	0.958 (0.115)	0.475^a^				
Short-run High GLE	719.368 (390.961)	982.643 (415.463)	< 0.001^a^	> 693.117	89.2	46.2	0.697
Short-run Low GLE (× 10^–3^)	2.294 (1.096)	1.730 (0.997)	< 0.001^a^	≤ 1.993	68.9	64.1	0.675
*Second order—neighborhood gray tone difference matrix (NGTDM)*							
Busyness	27.061 (27.856)	35.585 (28.361)	0.071^a^				
Coarseness (× 10^–5^)	2.331 (2.957)	1.785 (1.479)	0.007^a^	≤ 2.505	75.7	47.9	0.616
Complexity	5736.34 ± 1210.86	6093.04 ± 1887.26	0.150^b^				
Contrast	0.2082 ± 0.0766	0.2329 ± 0.0694	0.026^b^	> 0.2012	66.2	52.1	0.605
Strength (× 10^–2^)	3.518 (3.577)	2.365 (1.710)	< 0.001^a^	≤ 2.629	63.5	66.7	0.655
*Second order—gray level size zone matrix (GLSZM)*							
GL NUN (× 10^– 2^)	2.810 (0.567)	2.615 (0.299)	< 0.001^a^	≤ 2.748	73.0	55.6	0.663
GL Variance	103.41 ± 21.82	110.81 ± 20.47	0.020^a^	> 97.76	79.7	43.6	0.610
High GL Zone Emphasis	816.41 (376.85)	1057.04 (340.56)	< 0.001^b^	> 873.41	77.0	59.0	0.700
Low GL Zone Emphasis(× 10^–2^)	2.429 (1.146)	1.775 (0.880)	< 0.001^a^	≤ 2.234	74.3	56.4	0.677
Size Zone NUN	0.4399 (0.0395)	0.4425 (0.0499)	0.884^b^				
Large Area High GLE	16,758 (42,765)	25,675 (86,414)	0.033^a^	> 11,560	85.1	37.6	0.592
Large Area Low GLE	0.0964 (0.3851)	0.0635 (0.2388)	0.229^a^				
Small Area High GLE	620.68 ± 186.50	745.62 ± 203.19	< 0.001^b^	> 616.24	74.3	58.1	0.689
Small Area Low GLE (× 10^–3^)	1.642 (0.790)	1.231 (0.627)	< 0.001^a^	≤ 1.773	87.8	41.9	0.675
Zone Entropy	7.1386 ± 0.1490	7.2035 ± 0.1828	0.008^b^	> 7.2178	59.5	71.8	0.639
Zone Percentage	0.4554 ± 0.0678	0.4623 ± 0.0806	0.527^b^				
Zone Variance	26.686 (86.459)	29.728 (160.183)	0.981^a^				
*Second order—gray leve dependence matrix (GLDM)*							
Dependence Entropy	7.410 ± 0.179	7.511 ± 0.194	< 0.001^b^	> 7.489	62.2	69.2	0.666
Dependence Nonuniformity	85,241 (96,288)	138,809 (115,232)	0.001^a^	> 94,982	74.3	55.6	0.646
Dependence NUN	0.2321 (0.0407)	0.2359 (0.0477)	0.314^b^				
Dependence variance	2.077 (0.815)	1.983 (0.776)	0.281^a^				
Dependence GLNU	13,166 (15,270)	17,013 (18,239)	0.031^a^	> 15,837	58.1	59.8	0.593
GL Variance	95.750 ± 28.192	106.284 ± 25.285	0.010^b^	> 95.667	73.0	53.0	0.618
High GLE	834.296 (424.553)	1047.474 (442.943)	< 0.001^a^	> 719.576	89.2	47.9	0.698
Low GLE (× 10^–3^)	2.408 (1.157)	1.801 (1.059)	< 0.001^a^	≤ 2.126	68.9	63.2	0.672
Large Dependence Emphasis	8.245 (2.248)	8.690 (2.938)	0.419^a^				
Small Dependence Emphasis	0.397 ± 0.055	0.403 ± 0.067	0.534^b^				
Large DHGLE	5541.21 (2636.73)	7369.43 (3673.84)	< 0.001^a^	> 6562.75	70.3	58.4	0.708
Large DLGLE (× 10^– 2^)	1.979 (2.010)	1.388 (1.199)	0.001^a^	≤ 1.455	59.5	68.4	0.646
Small DHGLE	354.751 ± 134.628	435.646 ± 147.741	< 0.001^b^	> 331.553	78.4	53.0	0.670
Small DLGLE (× 10^– 4^)	8.898 (4.828)	6.843 (3.277)	< 0.001^a^	≤ 8.198	68.9	62.4	0.678

Group results were given as Median (IQR) or Mean ± SD according to the parameter’s Nonnormal or Normal distribution, respectively

Group1: Patients discharged from inpatient floor; Group 2: Patients transferred to ICU

*IDN* inverse difference normalized, *IMC* informational measure of correlation, *RMAD* robust mean absolute deviation, *GL* gray level, *GLE* gray level emphasis, *NUN* nonuniformity normalized, *DHGLE* dependence high gray level emphasis, *DLGLE* dependence low gray level emphasis

^*^Standardized results

^a^Mann–Whitney test result

^b^T-test result

^c^ROC analysis result

### Predictive logit-fit models

None of the clinical, volumetric or radiomics parameters provided a dependable univariate classifier. Therefore, logit-fit models were created (Table 5).

**Table 5:** Features of clinical, radiological and combined models in detecting ICU candidate COVID-19 patients

Model features				Training and validation set**	Test set***					
Type	Parameters	*p*	Odds Ratio* (95% CI)	C-index 95% CI^§^	AUC	Spec%	Sens%	Acc%	Precision	F1
Clinical model	Age	0.002	1.057 (1.023–1.092)	Training 0.846 0.824–0.866 Validation 0.805 0.724–0.869	0.736	79.17	66.67	74.36	66.67	0.67
	Platelet count	0.117	0.995 (0.990–0.999)							
	eGFR	0.385	0.989 (0.966–1.014)							
	AST	0.322	1.016 (0.998–1.034)							
	LDH	< 0.001	1.007 (1.003–1.011)							
	Procalcitonin	< 0.001	8.542 (1.892–38.559)							
	Fever > 38 °C	0.092	3.418 (1.439–8.121)							
Radiological model	Percent infected lung	0.010	1.043 (1.009–1.078)	Training 0.841 0.819–0.860 Validation 0.815 0.746–0.877	0.708	79.17	60.00	71.79	64.28	0.62
	First order skewness	< 0.001	0.029 (0.006–0.138)							
	First order RMAD	0.030	0.969 (0.942–0.997)							
	GLCM sumentropy	0.002	7.181 (1.974–26.128)							
	GLDM LDLGLE	0.005	5.486 (1.650–18.245)							
Combined model	Percent infected lung	0.015	1.048 (1009–1088)	Training 0.897 0.882–0.914 Validation 0.853 0.787–0.905	0.794	87.50	73.33	82.05	78.57	0.76
	First order skewness	0.001	0.012 (0.001–0.166)							
	GLCM clustershade	0.058	1.000 (1.000–1.001)							
	GLSZM LALGLE	0.002	1.525 (1.174–1.982)							
	N/L ratio	0.051	1.088 (1.000–1.184)							
	Platelet count	0.009	0.993 (0.987–0.998)							
	eGFR	< 0.001	0.962 (0.942–0.983)							
	AST	0.024	1.021 (1.003–1.040)							

*AUC* area under curve, *Spec%* specificity %, *Sens%* sensitivity % (Recall), *Acc%* accuracy %, *eGFR* estimated glomerular filtration rate, *AST* aspartate aminotransferase, *LDH* lactate dehydrogenase, *N/L* neutrophil to lymphocyte ratio, *GLCM* gray level co-occurrence matrix, *GLDM LDLGLE* gray level dependence matrix LargeDependenceLowGrayLevelEmphasis, *GLSZM LALGLE* gray level size zone matrix LargeAreaLowGrayLevelEmphasis

*Standardized values, **Cut-off = 0.500, ***Model-specific cut-off, **§** 95% CI calculated via 1000 iterations of bootstrap resampling

The clinical model’s PP was calculated using Eq. (2):

2 $$\begin{aligned} {\text{PP}} & = {1}/({1} + {\text{exp}}{-}({-}{5}.{18}0 + 0.0{46} \times {\text{Age}}{-}0.00{41} \times {\text{PLT}}{-}0.0{1}0 \times {\text{GFR}} \\ & \quad + 0.0{11} \times {\text{AST}} + 0.00{8} \times {\text{LDH}} + {2}.{556} \times {\text{PCT}} + 0.{787} \times {\text{Fever}})) \\ \end{aligned}$$

where PLT is the platelet count, GFR is the estimated glomerular filtration rate, AST is aspartate aminotransferase, LDH is lactate dehydrogenase and PCT is procalcitonin. Although the clinical model had good specificity, its sensitivity was limited in the training and validation sets (Table 5).

Radiological model’s PP calculated using Eq. (3):

3 $$\begin{aligned} {\text{PP}} & = {1}/({1} + {\text{exp}}{-}({-}{48}.{219} + 0.0{45} \times {\text{PIL}}{-}0.0{3}0 \times {\text{RMAD}} - {3}.{664} \\ & \quad \times {\text{Skewness}} + {8}.{532} \times {\text{GLCM Sumentropy}} + {82}.{32}0 \times {\text{LDLGLE}})) \\ \end{aligned}$$

where PIL is the percent of infected lung, RMAD is Robust Mean Absolute Deviation, and LDLGLE is GLDM-LargeDependenceLowGrayLevelEmphasis. This model showed a better sensitivity but a worse specificity than the clinical model (Table 5).

Combined model’s PP calculated using Eq. (4):

4 $$\begin{aligned} {\text{PP}} & = {1}/({1} + {\text{exp}}{-}({2}.{633} + 0.0{51} \times {\text{PIL}}{-}{4}.{596} \times {\text{S}} + 0.000{3}0{3} \times {\text{C}} + 0.{435} \\ & \quad \times {\text{GLE}} + 0.0{85} \times {\text{NLR}}{-}0.00{7} \times {\text{PLT}}{-}0.0{39} \times {\text{GFR}} + 0.0{2}0 \times {\text{AST}})) \\ \end{aligned}$$

where PIL is the percent of infected lung, S is the skewness, C is GLCM-Clustershade, GLE is GLSZM-LargeAreaLowGrayLevelEmphasis, NLR is the neutrophil-to-lymphocyte ratio, PLT is the platelet count, GFR is the estimated glomerular filtration rate, and AST is aspartate aminotransferase. This model had the highest AUC and specificity (Table 5).

**Fig.2:**
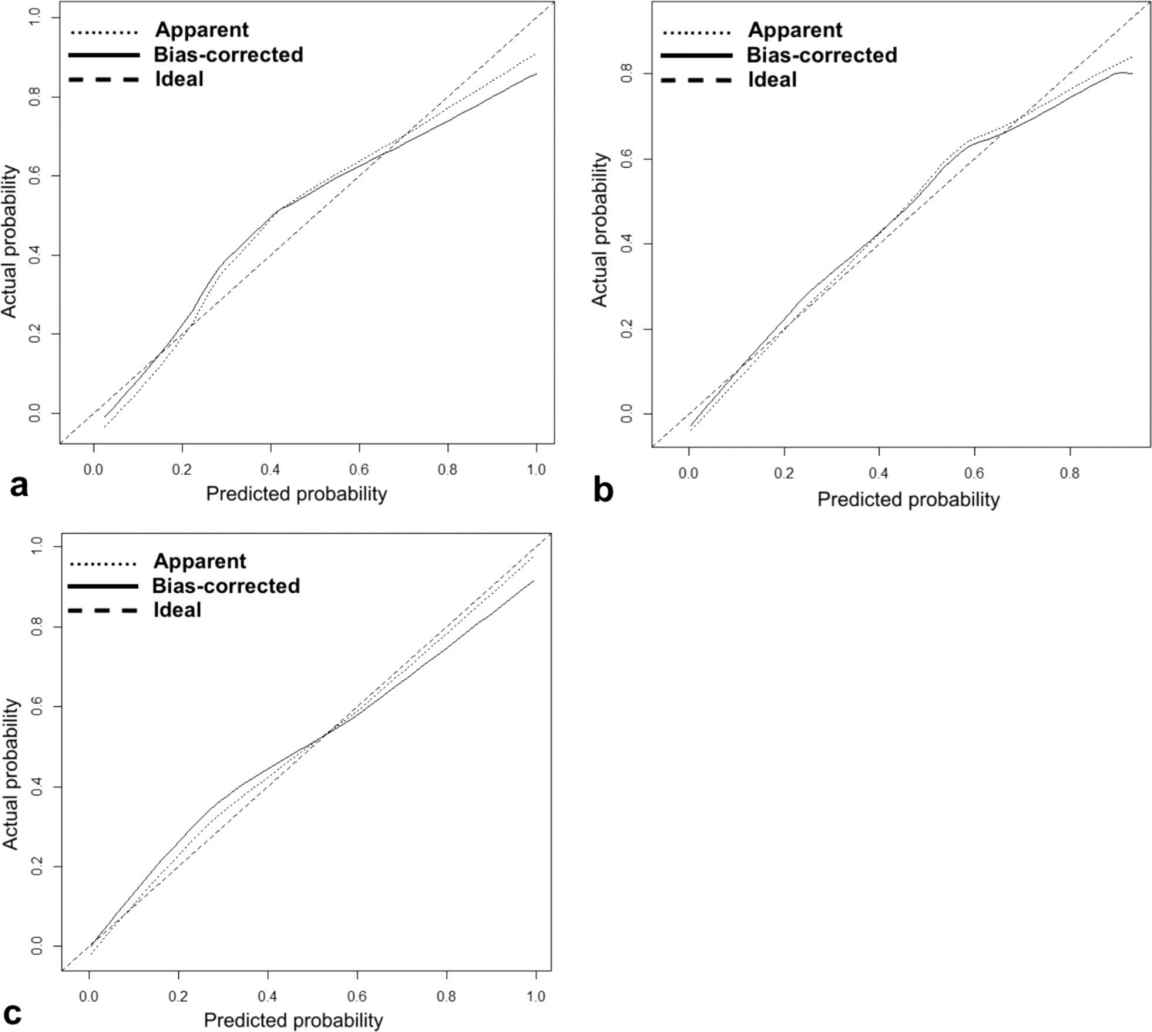
Calibration plot of Clinical (**a**), Radiological (**b**), and Combined (**c**) models

The calculated *p* values for the clinical, radiological, and combined models were 0.376, 0.399, and 0.631, respectively, in the Hosmer–Lemeshow test. Radiologic and combined models showed better calibration than the clinical model (Fig. 2). The VIF value was less than 3.0 for all parameters in the models; thus, there was no significant multicollinearity.

**Fig.3:**
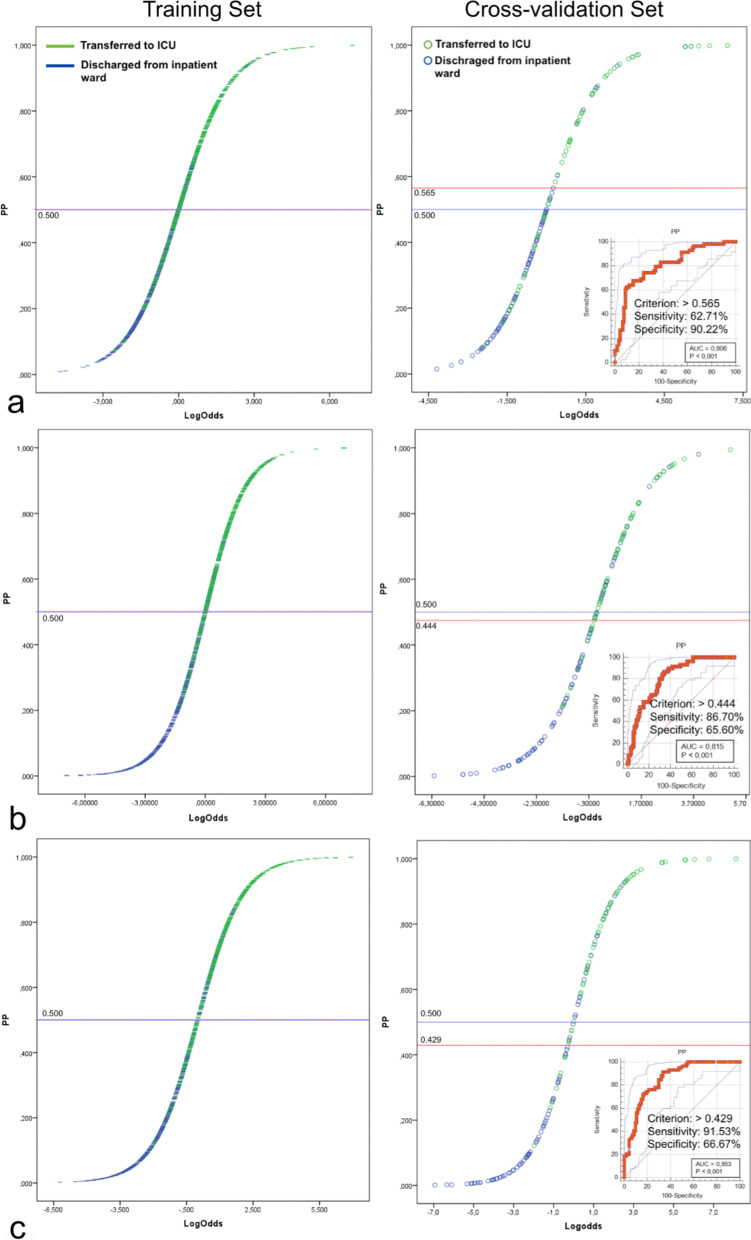
Prediction probability score versus model score graph of clinical (**a**), radiological (**b**), and combined (**c**) models. In the ROC analysis of the cross-validation sets, the optimal cutoff values of the models were determined and marked by using the Youden index

The optimal cutoff-off values were calculated for the clinical, radiological, and combined models as 0.565, 0.444, and 0.429, respectively (Fig. 3).

### Test set features and test set results of logit-fit models

Fifty-nine patients in the training set and 15 in the test set were transferred to the ICU (*p* = 0.908, chi-squared test). The median age of the patients was 65 (22.8) years in the training set and 61 [20] years in the test set (*p* = 0.056, Mann–Whitney test). The training set included 88 males and 64 females, and the test set included 22 males and 17 females (*p* = 0.867, chi-squared test).

In the test set, the combined model produced the best AUC, followed by the clinical model (Table 5).

### Synthetic data generation and random forest algorithm

Despite the fact that they were well calibrated, there were problems with the logit-fit models due to the study population. The data had a low sample size and were affected by class-imbalance. Due to the fact that our sample size was low, the BIC method, which penalizes complex models with more parameters [25], was preferred in the model selection for logit-fit models to avoid overfitting [26]. In addition to the logit-fit models, a random forest classification algorithm, as a method that resistant to overfitting, was used for generating a model that uses all of the study parameters.

While 93 (61%) patients in the training set did not require intensive care, 59 (39%) patients were transferred to the ICU. The use of the unbalanced training set, especially for the high-dimensional data, was reported as a reason for model bias in favor of the majority class [27]. To solve this problem, the instance of the minority class, which involves patients being transferred to the ICU, was increased to 93 by using the SMOTE algorithm.

The prediction results on the test set of the model that was trained with the random forest algorithm on a more balanced training set did not lead to an increase in specificity (87.5%). On the other hand, sensitivity (80%) was increased, and increases in accuracy (84.6%), AUC (0.837), precision (0.80), and F1 score (0.80) were followed.

**Fig.4:**
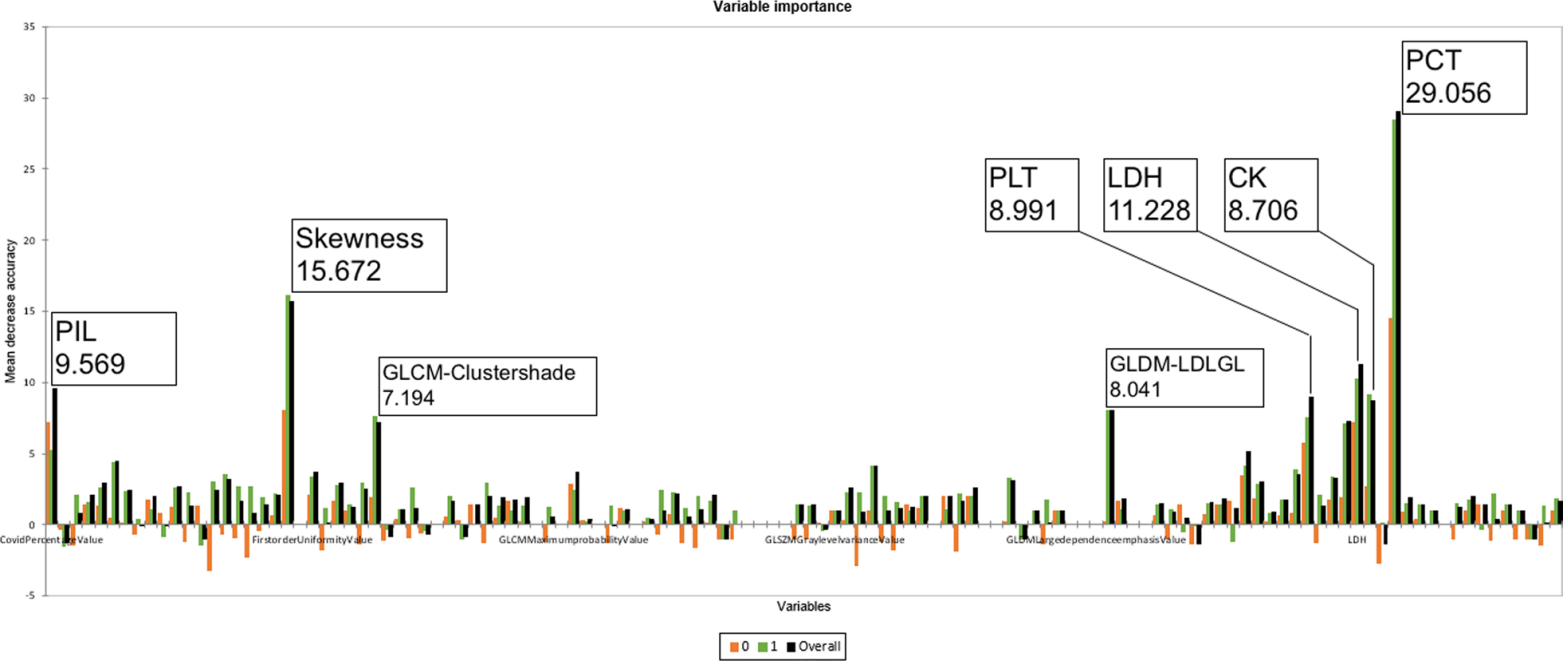
Mean Decrease Accuracy of study parameters in Random Forest model. Features with the highest overall importance were indicated. PIL: Percent infected lung, PLT: Platelet count, LDH: Lactate dehydrogenase, CK: Creatine kinase, PCT: Procalcitonin

A feature importance study was also conducted for the random forest model (Fig. 4). Overall importance was the highest in the PCT, followed by Skewness, LDH, PIL, CK, and GLDM-LargeDependenceLowGrayLevelEmphasis. It was observed that most of the parameters that were included in the logit-fit models also had high mean decrease accuracy values in the random forest model.

**Fig. 5:**
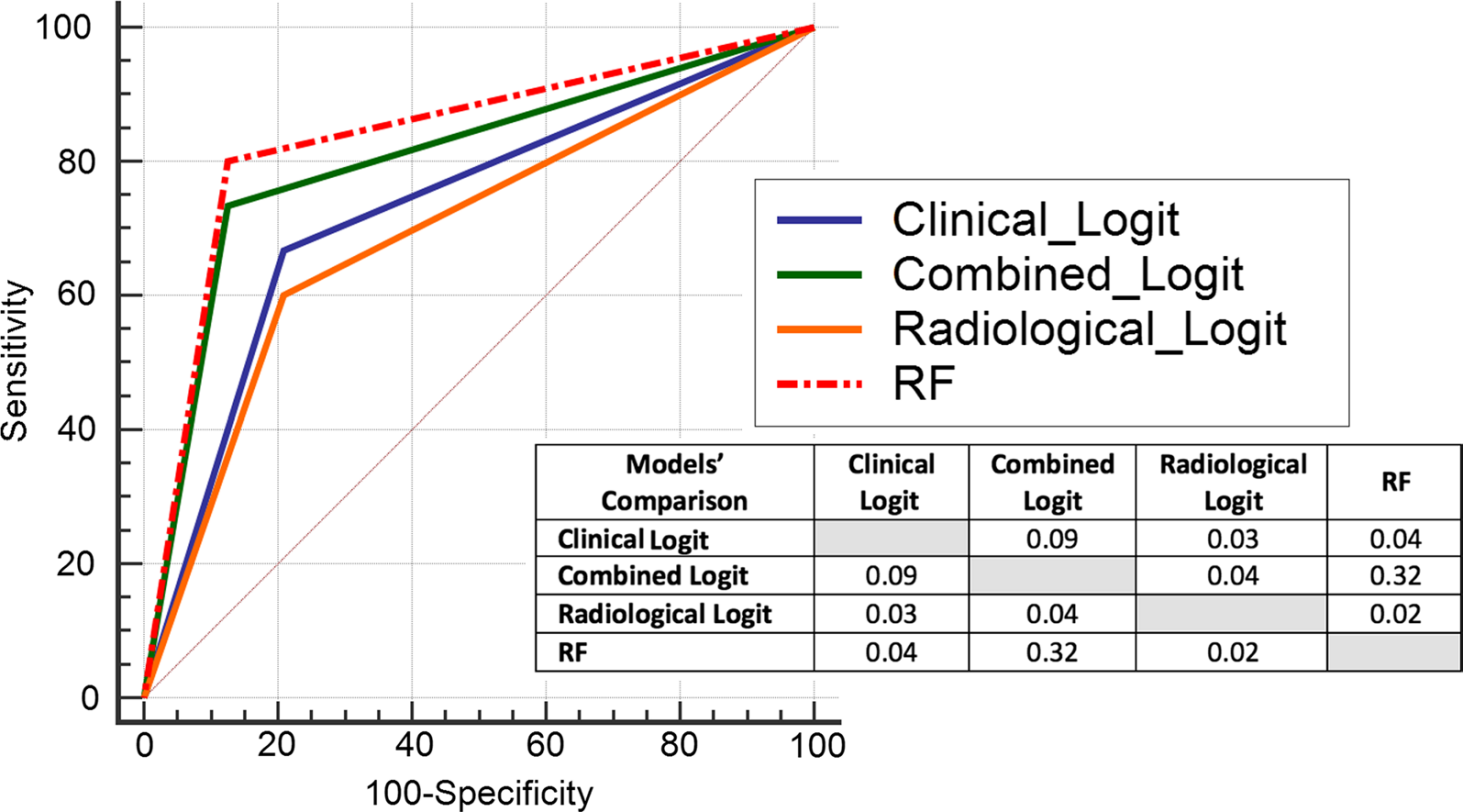
ROC curves and their non-parametric pairwise comparison table of machine learning models in the study. The *p* values of the comparison results were given. RF: Random Forest model

When the ROC curves of the models were evaluated by pairwise comparison [28], no significant difference was found between the RF model and the Combined logit fit model (Fig. 5).

### Power analysis

On the post hoc analysis, for a difference of the mean for two independent groups including 117 and 74 patients with input parameters of a medium effect size (Cohen’s d = 0.5), two tails, and alpha = 0.05, the calculated power was 0.91.

## Discussion

COVID-19 surges in the United States over the past two years were assessed by the CDC as three periods [29]. These are the Winter 2020–2021 period, the Delta period from July to November 2021, and the Omicron period that we are currently in, which started in December 2021. The maximum number of 7-day moving average ICU bed in use for COVID-19 was reported as 27,958 (January 9–16, 2021) in the Winter 2020–2021 period, 24,775 (September 6–13, 2021) in the Delta period, and 24,776 (January 15, 2022) in the Omicron period [29]. It has been reported that the number of patients who required intensive care due to the Omicron variant is one-fourth compared to the Delta variant [30]. The lack of difference between the ICU admission numbers between the Omicron period and the Delta period is due to the difference in the number of COVID-19 cases. While the maximum case number of 7-day moving average in the Delta period was 164,249, this value was reported as 798,976 in the Omicron period [29].

Since none of the 28 clinical, 21 volumetric, and 74 radiomics parameters could reliably predict patients who would require ICU admission, clinical, radiological, and combined models were built, and the combined model provided the best predictions.

Models based solely on the clinical data emerged with easy accessibility and usability features. High fever, older age, elevated LDH, increased acute phase reactants and a decreased lymphocyte count were frequently reported in ICU candidates [4–11]. In our study, procalcitonin was distinguished as the parameter with the highest odds ratio value, and patients with chronic renal failure showed a significantly higher need for ICU care, which is consistent with previous publications [12].

In a study involving cases from 100 hospitals in South Korea, the presence or absence of chest X-ray findings did not significantly improve the clinical model outcomes when used as a parameter [12]. On the other hand, it has been reported that a better prediction was obtained when a deep-learning model, which was trained to discriminate critical and noncritical chest X-ray findings, was combined with a clinical model [31].

In prognostic studies that have evaluated CT findings, both increased volumes in pulmonary involvement [32] and an increased ratio of consolidation in pulmonary lesions [33] were associated with an unfavorable prognosis. In our study, the percentage of infected lung parenchyma was included in the radiological and combined models. The mean and median densities of the lesions were significantly higher in ICU patients, suggesting a higher frequency of consolidation. However, this parameter was not included, and skewness was selected for the models by BIC. It has been previously shown that as the GGO areas in the lesions increase, the skewness value of the lesion also increases [18]. We showed that the skewness of the lesions was significantly higher in patients who did not require ICU admission. VSS, which includes the classification of lesions as GGO or consolidation, has been reported as an effective method in the evaluation of COVID-19 prognosis [34, 35]. However, this method is reported to have reliability and reproducibility problems due to issues such as difficulty classifying lesions containing both areas of consolidation and GGO, and radiomics models were found to be more useful for predicting prognosis [36].

The total lung volume was 12% lower in the ICU group. Alveolar collapse is known to occur in patients with SARS-CoV-2 infection [37, 38], and surfactant reduction that results from the loss of alveolar type 2 cells, increased inflammatory cell migration to the interstitial space, and microvascular thrombosis may be responsible for this outcome [39]. Although the total lung volume was not directly entered into the models in the parameter selections, it participated in the calculation of the percent of infected lung parameter.

Although segmentation can be performed manually in radiomics modeling studies related to COVID-19 [18, 40], this method takes considerable time due to the large number of lesions per patient, and the reproducibility problem needs to be overcome. Methods such as the segmentation of the entire lung (healthy and diseased), rather than individual lesions, have been suggested [36].

Automated segmentation solves all of these problems. While the use of AI in CT sections is not recommended as a screening test, its use as a predictive and prognostic decision support system in hospitalized patients has been suggested [41]. In a study examining clinical data and the radiomic features calculated from automated segmentations, a combination model produced the best predictions [19]. In our study, apart from radiomic parameters, the percent of infected lung parameter was included in the models. Thus, the subjective calculation of critical parameters, such as the lesion classification and the ratio of the diseased parenchyma in the VSS method, were solved, and the models were based on objective criteria. Additionally, volumetric parameters produced by automated segmentations are reportedly more accurate than human semiquantitative estimates [42].

The model that we propose for predicting the risk of ICU in the COVID-19 patient has two important features. First, it does not solely use clinical data. Models that are solely based on laboratory parameters did not consider lung parenchyma involvement as a parameter, nevertheless all of the combined models in our study included more than one radiological parameter, regardless of the machine learning algorithm that was used. Second, the reproducibility problem of VSS methods has been resolved by using the segmentations of the deep learning algorithm that was trained with CT studies from multiple hospitals in affected countries across Europe. Thus, we believe that models based on non-subjective clinical and radiological data that require no parameter calculation effort and that provide reproducible results could be more widely used in the field and can help healthcare providers to make decisions and better organize hospitals' resources.

This study has some limitations. First, these are the results of single center. However, we used automated segmentation, and CT data were resampled during the radiomics parameter calculation. Second, the patient population was retrospectively selected from patients who had an indication for hospitalization, which could introduce selection bias. Third, the relationship between the antiviral treatment efficacy and the ICU requirements was not evaluated in this study since there is no definitively proven antiviral treatment for COVID-19. Fourth, we used an unbalanced data set; however, we increased the sensitivity by adding synthetic data to the training set. Finally, patients with a contrast-enhanced examination were not included in this study since the radiomics parameters would be affected. We believe that a different model is required for embolism cases.

## Conclusion

The model that was created by combining the radiological parameters obtained by automated segmentation and the clinical parameters in COVID-19 patients requiring hospitalization was found to be useful as an objective method in predicting the risk of developing critical illness.

## Data Availability

All data generated or analyzed during this study are included in this published article.

## Abbreviations

AUC: Area under curve
DCA: Decision curve analysis
DL: Deep learning
FiO_2_: Fraction of inspired oxygen
GGO: Ground glass opacities
GLCM: Gray level co-occurrence matrix
GLDM: Gray level dependence matrix
GLRLM: Gray level run-length matrix
GLSZM: Gray level size zone matrix
IQR: Interquartile range
NLR: Neutrophil to lymphocyte ratio
PaO2: Partial pressure of oxygen
RMAD: Robust mean absolute deviation
ROC: Receiver operator characteristics
SD: Standard deviation
SMOTE: Synthetic Minority Oversampling TEchnique
SpO2: Oxygen saturation (estimated by pulse oximetry)
VIF: Variance inflation factor
VSS: Visual severity score

## References

[1] COVID-19 Treatment Guidelines Panel. Coronavirus disease 2019 (COVID-19) treatment guidelines. National Institutes of Health. Available at https://www.covid19treatmentguidelines.nih.gov/. Accessed 24 Oct 2021. 34003615

[2] Goic M, Bozanic-Leal MS, Badal M (2021). COVID-19: short-term forecast of ICU beds in times of crisis. PLoS ONE.

[3] Pascarella G, Strumia A, Piliego (2020). COVID-19 diagnosis and management: a comprehensive review (review). J Intern Med.

[4] Ayaz A, Arshad A, Malik H (2020). Risk factors for intensive care unit admission and mortality in hospitalized COVID-19 patients. Acute Crit Care.

[5] Vanhems P, Gustin MP, Elias C (2021). Factors associated with admission to intensive care units in COVID-19 patients in Lyon-France. PLoS ONE.

[6] Gong J, Ou J, Qiu X (2020). A tool to early predict severe corona virus disease 2019 (COVID-19): a multicenter study using the risk nomogram in Wuhan and Guangdong. China Clin Infect Dis.

[7] Yan L, Zhang H-T, Xiao Y, Wang M, Sun C, Liang J, et al. Prediction of criticality in patients with severe Covid-19 infection using three clinical features: a machine learning-based prognostic model with clinical data in Wuhan. medRxiv. 2020. 10.1101/2020.02.27.20028027.

[8] Liang W, Liang H, Ou L, Chen B, Chen A, Li C (2020). Development and validation of a clinical risk score to predict the occurrence of critical illness in hospitalized patients with COVID-19. JAMA Intern Med.

[9] Caramelo F, Ferreira N, Oliveiros B. Estimation of risk factors for COVID-19 mortality - preliminary results. medRxiv. 2020. 10.1101/2020.02.24.20027268.

[10] Ji D, Zhang D, Xu J (2020). Prediction for progression risk in patients with COVID-19 pneumonia: the CALL score. Clin Infect Dis.

[11] Xie J, Hungerford D, Chen H, Development and external validation of a prognostic multivariable model on admission for hospitalized patients with COVID-19. medRxiv. 2020. 10.1101/2020.03.28.20045997.

[12] Heo J, Han D, Kim HJ (2021). Prediction of patients requiring intensive care for COVID-19: development and validation of an integer-based score using data from Centers for Disease Control and Prevention of South Korea. J Intensive Care.

[13] Liu S, Nie C, Xu Q (2021). Prognostic value of initial chest CT findings for clinical outcomes in patients with COVID-19. Int J Med Sci.

[14] Wasilewski PG, Mruk B, Mazur S, Półtorak-Szymczak G, Sklinda K, Walecki J (2020). COVID-19 severity scoring systems in radiological imaging: a review. Pol J Radiol.

[15] Yu Q, Wang Y, Huang S (2020). Multicenter cohort study demonstrates more consolidation in upper lungs on initial CT increases the risk of adverse clinical outcome in COVID-19 patients. Theranostics.

[16] Gillies RJ, Kinahan PE, Hricak H (2016). Radiomics: images are more than pictures. They Are Data Radiol.

[17] Zhang X, Wan D, Shao J (2021). A deep learning integrated radiomics model for identification of coronavirus disease 2019 using computed tomography. Sci Rep.

[18] Gülbay M, Özbay BO, Mendi BA (2021). A CT radiomics analysis of COVID-19-related ground-glass opacities and consolidation: Is it valuable in a differential diagnosis with other atypical pneumonias?. PLoS ONE.

[19] Wang D, Huang C, Bao S (2021). Study on the prognosis predictive model of COVID-19 patients based on CT radiomics. Sci Rep.

[20] Wu Q, Wang S, Li L (2020). Radiomics analysis of computed tomography helps predict poor prognostic outcome in COVID-19. Theranostics.

[21] Huang S, Yang J, Fong S (2021). Artificial intelligence in the diagnosis of COVID-19: challenges and perspectives. Int J Biol Sci.

[22] Saood A, Hatem I (2021). COVID-19 lung CT image segmentation using deep learning methods: U-Net versus SegNet. BMC Med Imaging.

[23] Taylor S. Logistic regression: application to clinical classification. UC Davis Health—Clinical and Translational Science Center. https://health.ucdavis.edu/ctsc/area/Resource_Library/documents/LogisticRegression_II_10March2021.pdf. Accessed 24 Oct 2021.

[24] Package ‘smotefamily’. https://cran.r-project.org/web/packages/smotefamily/smotefamily.pdf. Accessed 23 Jan 2022.

[25] Bayesian information criterion. https://en.wikipedia.org/wiki/Bayesian_information_criterion. Accessed 23 Jan 2022.

[26] Peduzzi P, Concato J, Kemper E (1996). A simulation study of the number of events per variable in logistic regression analysis. J Clin Epidemiol.

[27] Blagus R, Lusa L (2013). SMOTE for high-dimensional class-imbalanced data. BMC Bioinform.

[28] DeLong ER, DeLong DM, Clarke-Pearson DL (1988). Comparing the areas under two or more correlated receiver operating characteristic curves: a nonparametric approach. Biometrics.

[29] Trends in Disease Severity and Health Care Utilization During the Early Omicron Variant Period Compared with Previous SARS-CoV-2 High Transmission Periods—United States, December 2020–January 2022. https://www.cdc.gov/mmwr/volumes/71/wr/mm7104e4.htm#F1_down. Accessed 26 Mar 2022. 10.15585/mmwr.mm7104e4 PMC9351529 35085225

[30] Abdullah F, Myers J, Basu D (2022). Decreased severity of disease during the first global omicron variant covid-19 outbreak in a large hospital in Tshwane, South Africa. Int J Infect Dis.

[31] Jiao Z, Choi JW, Halsey K (2021). Prognostication of patients with COVID-19 using artificial intelligence based on chest x-rays and clinical data: a retrospective study. Lancet Digit Health.

[32] Yuan M, Yin W, Tao Z (2020). Association of radiologic findings with mortality of patients infected with 2019 novel coronavirus in Wuhan, China. PLoS ONE.

[33] Cau R, Falaschi Z, Paschè A (2021). Computed tomography findings of COVID-19 pneumonia in Intensive Care Unit-patients. J Public Health Res.

[34] Wang X, Hu X, Tan W (2021). Multicenter study of temporal changes and prognostic value of a CT visual severity score in hospitalized patients with coronavirus disease (COVID-19). Am J Roentgenol.

[35] Zhao W, Zhong Z, Xie X (2020). Relation between chest CT findings and clinical conditions of coronavirus disease (COVID-19) pneumonia: a multicenter study. Am J Roentgenol.

[36] Homayounieh F, Ebrahimian S, Babaei R (2020). CT radiomics, radiologists, and clinical information in predicting outcome of patients with COVID-19 pneumonia. Radiol Cardiothorac Imaging.

[37] Iwasawa T, Sato M, Yamaya T (2020). Ultra-high-resolution computed tomography can demonstrate alveolar collapse in novel coronavirus (COVID-19) pneumonia. Jpn J Radiol.

[38] Shi F, Wei Y, Xia L (2021). Lung volume reduction and infection localization revealed in Big data CT imaging of COVID-19. Int J Infect Dis.

[39] Savaş R, Öz ÖA (2021). Evaluation of lung volume loss with 3D CT volumetry in COVID-19 patients. Diagn Interv Radiol.

[40] Wang L, Kelly B, Lee EH (2021). Multi-classifier-based identification of COVID-19 from chest computed tomography using generalizable and interpretable radiomics features. Eur J Radiol.

[41] Neri E, Miele V, Coppola F (2020). Use of CT and artificial intelligence in suspected or COVID-19 positive patients: statement of the Italian Society of Medical and Interventional Radiology. Radiol Med.

[42] Kanne JP, Bai H, Bernheim A (2021). COVID-19 imaging: what we know now and what remains unknown. Radiology.

